# Evaluation of Spending Differences Between Beneficiaries in Medicare Advantage and the Medicare Shared Savings Program

**DOI:** 10.1001/jamanetworkopen.2022.28529

**Published:** 2022-08-23

**Authors:** Ravi B. Parikh, Ezekiel J. Emanuel, Colleen M. Brensinger, Connor W. Boyle, Eboni G. Price-Haywood, Jeffrey H. Burton, Sabrina B. Heltz, Amol S. Navathe

**Affiliations:** 1 Perelman School of Medicine, University of Pennsylvania, Philadelphia; 2 Leonard Davis Institute of Health Economics, University of Pennsylvania, Philadelphia; 3 Corporal Michael J. Crescenz VA Medical Center, Philadelphia, Pennsylvania; 4 Ochsner Health Network, New Orleans, Louisiana

## Abstract

**Importance:**

The 2 primary efforts of Medicare to advance value-based care are Medicare Advantage (MA) and the fee-for-service–based Medicare Shared Savings Program (MSSP). It is unknown how spending differs between the 2 programs after accounting for differences in patient clinical risk.

**Objective:**

To examine how spending and utilization differ between MA and MSSP beneficiaries after accounting for differences in clinical risk using data from administrative claims and electronic health records.

**Design, Setting, and Participants:**

This retrospective economic evaluation used data from 15 763 propensity score–matched beneficiaries who were continuously enrolled in MA or MSSP from January 1, 2014, to December 31, 2018, with diabetes, congestive heart failure (CHF), chronic kidney disease (CKD), or hypertension. Participants received care at a large nonprofit academic health system in the southern United States that bears risk for Medicare beneficiaries through both the MA and MSSP programs. Differences in beneficiary risk were mitigated by propensity score matching using validated clinical criteria based on data from administrative claims and electronic health records. Data were analyzed from January 2019 to May 2022.

**Exposures:**

Enrollment in MA or attribution to an accountable care organization in the MSSP program.

**Main Outcomes and Measures:**

Per-beneficiary annual total spending and subcomponents, including inpatient hospital, outpatient hospital, skilled nursing facility, emergency department, primary care, and specialist spending.

**Results:**

The sample of 15 763 participants included 12 720 (81%) MA and 3043 (19%) MSSP beneficiaries. MA beneficiaries, compared with MSSP beneficiaries, were more likely to be older (median [IQR] age, 75.0 [69.9-81.8] years vs 73.1 [68.3-79.8] years), male (5515 [43%] vs 1119 [37%]), and White (9644 [76%] vs 2046 [69%]) and less likely to live in low-income zip codes (2338 [19%] vs 750 [25%]). The mean unadjusted per-member per-year spending difference between MSSP and MA disease-specific subcohorts was $2159 in diabetes, $4074 in CHF, $2560 in CKD, and $2330 in hypertension. After matching on clinical risk and demographic factors, MSSP spending was higher for patients with diabetes (mean per-member per-year spending difference in 2015: $2454; 95% CI, $1431-$3574), CHF ($3699; 95% CI, $1235-$6523), CKD ($2478; 95% CI, $1172-$3920), and hypertension ($2258; 95% CI, $1616-2,939). Higher MSSP spending among matched beneficiaries was consistent over time. In the matched cohort in 2018, MSSP total spending ranged from 23% (CHF) to 30% (CKD) higher than MA. Adjusting for differential trends in coding intensity did not affect these results. Higher outpatient hospital spending among MSSP beneficiaries contributed most to spending differences between MSSP and MA, representing 49% to 62% of spending differences across disease cohorts.

**Conclusions and Relevance:**

In this study, utilization and spending were consistently higher for MSSP than MA beneficiaries within the same health system even after adjusting for granular metrics of clinical risk. Nonclinical factors likely contribute to the large differences in MA vs MSSP spending, which may create challenges for health systems participating in MSSP relative to their participation in MA.

## Key Points

**Question:**

How do spending and utilization differ between Medicare Advantage (MA) and Medicare Shared Savings Program (MSSP) beneficiaries after accounting for clinical risk in a health system participating in both programs?

**Findings:**

In this economic evaluation of 15 763 MA and MSSP beneficiaries between 2014 and 2018, spending was 22% to 26% higher for MSSP beneficiaries than for MA beneficiaries even after controlling for detailed clinical risk factors. This was accounted for by higher outpatient hospital spending for MSSP beneficiaries.

**Meaning:**

In this study, spending differences between MA and MSSP beneficiaries persisted after accounting for granular clinical risk factors, suggesting the need for aligning program designs and accounting for unmeasured social determinants of health.

## Teaser

This economic evaluation examines how spending and utilization differ between Medicare Advantage and Medicare Sharer Savings Program beneficiaries after accounting for differences in clinical risk using data from administrative claims and electronic health records.

## Introduction

Two of Medicare’s key strategies to control cost growth and promote value-based care for its nearly 60 million beneficiaries are shared savings through the Medicare Shared Savings Program (MSSP) for traditional fee-for-service (FFS) Medicare and global capitation through Medicare Advantage (MA).^1,2^ The Patient Protection and Affordable Care Act required the Centers for Medicare & Medicaid Services (CMS) create a program to establish and reimburse accountable care organizations (ACOs). One of Medicare’s biggest ACO programs is the MSSP, which incentivizes cost containment by allowing participating health care organizations to share in savings to the Medicare program. The number of MSSP ACOs has steadily grown since 2012, and the overall number of MSSP beneficiaries represents approximately one-third of all FFS Medicare beneficiaries.^3^ Furthermore, a stated goal of the current administration’s CMS Innovation Center is to stimulate greater clinician and health care organization participation in ACOs such that every FFS beneficiary is attributed to one. MA has similarly grown rapidly, now constituting 34% of all Medicare beneficiaries, and is expected to represent more than 50% of Medicare beneficiaries by 2030.^4,5,6^ The MA program incentivizes cost containment by paying private insurers capitated, risk-adjusted payments from Medicare. In turn, the MA plan contracts with clinicians, hospitals, and health systems directly and often provides additional services and benefits to manage costs. Beneficiaries choose to enroll in MA based on plan offerings by geographic region. In contrast, beneficiaries are auto-enrolled in Medicare Part A for hospital coverage and can enroll in Part B for physician and nonfacility service coverage by submitting an application. These beneficiaries are assigned to MSSP by receiving primary care services within participating ACOs.

Understanding the incentives facing health organizations participating in MA and/or MSSP is critical to assessing the optimal policy options that can produce the highest cost containment and quality of care. Yet, making comparisons between MA and FFS using claims data is difficult because such data may not offer sufficient clinical granularity to account for meaningful differences in clinical risk of beneficiary characteristics.^7,8,9^ MA enrollees tend to be healthier than FFS enrollees, and beneficiaries with more severe illness may preferentially disenroll from MA.^10,11,12^ Even with similar practice and care patterns between MSSP and MA beneficiaries, systematically higher clinical risk may lead to higher spending and lower adherence to spending targets among MSSP beneficiaries compared with MA beneficiaries.^13^ This may further disincentivize health systems from participating in MSSP and other risk-based FFS models relative to MA. There have been important efforts to improve clinical risk adjustment in the MA program over the past 20 years.^14,15,16^ However, there may still be an inability to fully account for higher clinical risk among MSSP beneficiaries that may disincentivize health systems from participating in MSSP. Accounting for richer metrics of clinical risk factors from the electronic health record (EHR) could enable more accurate spending comparisons between MA and MSSP beneficiaries, with important implications for aligning incentives across programs.^17,18^

While the goal of this study was not to conduct program evaluations of MA and MSSP, our study used detailed EHR data to examine whether clinical risk accounts for spending differences between MA and MSSP. To examine this question, we studied spending patterns for MA and MSSP beneficiaries receiving care within the same large health system in the southern United States. We hypothesized that, after accounting for more granular metrics of clinical risk through the EHR and other factors such as area-level socioeconomic status and differences in coding intensity, there would be minimal spending differences between MA and MSSP beneficiaries.

## Methods

The study protocol was approved by the institutional review board at the University of Pennsylvania, including a waiver of informed consent for patients and physicians given the retrospective nature of the analysis. This economic evaluation followed the Consolidated Health Economic Evaluation Reporting Standards (CHEERS) 2022 guideline.

### Health System

Ochsner Health System (OHS) is a large, academic, nonprofit health system in Louisiana. OHS operates 40 hospitals and more than 100 health centers and urgent care clinics in Louisiana and Mississippi. In 2018, OHS provided care for 581 000 total patients and 145 000 Medicare beneficiaries. Since 2004, OHS has partnered with a large insurer to take full risk for beneficiaries covered by a MA plan. MA beneficiaries account for 58% of all Medicare beneficiaries at OHS and are generally responsible for 56% of total Medicare revenue. In 2013, OHS founded an ACO through the MSSP that consists of 2250 clinicians in Louisiana and Mississippi. As part of MSSP, OHS bears risk for FFS beneficiaries if it does not meet spending and quality benchmarks set by CMS. Since 2017, this ACO has consistently performed below spending benchmarks set by the MSSP program and received shared savings.

Through the MSSP program, OHS received complete Medicare FFS claims for beneficiaries in its patient population. Because OHS bore full risk for spending in the largest MA plan it contracts with, OHS also received complete MA claims data for these beneficiaries from the insurer. It is important to note that unlike MA encounter data acquired from CMS or other centralized sources, because these MA data were directly acquired from the insurer and OHS, they do not have the key limitations of MA encounter data, such as invalid prices and missing cost sharing data. These data included actual payments for services provided to allow for accurate spending calculations. This study only used data for these beneficiaries in MA and Medicare FFS for whom complete claims and actual payments were observed.

### Study Design

To avoid confounding from different patterns of care received, we identified beneficiaries with similar clinical characteristics between MSSP and MA. Our approach to identifying beneficiaries with similar clinical characteristics included selecting 4 common, high-cost, disease-specific cohorts for which evidence-based clinical risk-stratification algorithms exist (ie, hypertension, diabetes, congestive heart failure [CHF], and chronic kidney disease [CKD]) and matching individual beneficiaries across MA and MSSP cohorts using propensity scores. We also validated that practice patterns and quality of care did not markedly differ between MA and MSSP beneficiaries for these cohorts (eTable 1 and eFigure 1 in the Supplement). We examined the association between MA and MSSP enrollment status with spending, utilization, and quality outcomes when controlling for demographic characteristics, clinical variables, and socioeconomic variables. We used zip code fixed effects to account for time-invariant unobservable differences based on zip code of residence (eg, social determinants of health unobserved in the claims and clinical data) and primary care physician (PCP) fixed effects, thereby comparing MA and MSSP beneficiaries within the same PCP, to mitigate chances that outcome differences resulted from different clinician practice patterns.

### Study Periods

We used January 1, 2014, to December 31, 2018, as our study period. We defined the baseline period as January 1 to December 31, 2014. During the baseline period, claims and EHR data were used to build covariates for both unmatched and propensity-matched models. We defined a 4-year follow-up period as January 1, 2015, to December 31, 2018, during which we assessed spending and outcomes.

### Data Sources

Data for this study were obtained from OHS. We used Medicare claims to obtain demographic, Medicare eligibility, comorbidity, pharmacy, spending, and utilization data. We supplemented claims data with EHR data on laboratory values, vital signs, and other demographics from an internal OHS database.

### Study Population

Our study included beneficiaries who were continuously enrolled in the OHS MA and MSSP programs from 2014 through 2018. Enrollees were required to have continuous enrollment to the same plan across months for all 5 years of data unless the beneficiary died. Our cohort consisted of 15 763 Medicare enrollees who were alive on December 31, 2014; aged 18 years or older; were continuously enrolled in either MA or MSSP from January 2014 to December 2018; received primary care within the OHS; and had a diagnosis in 2014 of diabetes, CHF, CKD, or hypertension. eTable 2 in the Supplement presents criteria used to identify disease-specific cohorts. We excluded beneficiaries who were not continuously enrolled in either MA or MSSP plans; who switched from MA or MSSP (n = 435) or vice versa; or who received care from a PCP that was not affiliated with the OHS network.

### Exposure and Outcomes

The exposure was enrollment in the MA or attribution to an OHS ACO in the MSSP program. Outcomes were assessed per beneficiary between 2015 and 2018, stratified by year.

#### Spending

We measured annual total Medicare spending per beneficiary, defined as total OHS and non-OHS payments for all health care services. Secondary spending outcomes included spending for inpatient hospital, outpatient hospital, skilled nursing facility, emergency department, and primary care and specialist visits. Pharmacy spending was not examined because it was not available for a large proportion of MSSP beneficiaries, since prescription drug enrollment is not required for Medicare beneficiaries. All spending measures were adjusted for inflation to 2018 US dollars. To reduce the influence of some large outliers, all spending outcomes were winsorized at 1st and 99th percentiles.

#### Utilization

Utilization was a secondary outcome. We included number of inpatient hospitalizations, primary care visits, specialist visits, and emergency department visits.

### Covariates

Beneficiary-level covariates included age, sex, Charlson comorbidity index,^19^ and dual-eligibility status. We also linked beneficiary-level location data to area-level data from the American Community Survey wave 2011-2015 to determine the percentage of individuals in a beneficiary’s zip code of residence who were living below the poverty line. Area-level race and ethnicity data were collected to account for known racial/ethnic disparities in enrollment between MSSP and MA.

### Clinical Risk Indicators

We merged EHR data with Medicare claims data to extract data elements required to compute clinical risk based on established clinical risk stratification criteria.^20,21,22^ Variables consisted of demographic, laboratory, vital sign, and pharmacy markers of risk for each of the 4 diseases (eTable 3 in the Supplement ). For all laboratory and vital sign data, we used the last record value in 2014.

### Statistical Analysis

We compared baseline characteristics between the MA and MSSP cohorts using χ^2^ tests for categorical variables and Wilcoxon rank-sum tests for continuous variables. We used logistic regression to calculate propensity scores, defined as the estimated probability of being in MSSP vs the MA plan starting in 2015. The covariates included were based on 2014 data; they were generally grouped into demographic characteristics, clinical variables, and socioeconomic variables (eTable 3 in the Supplement). We matched MSSP to MA members (1:3) using nearest-neighbor caliper matching without replacement; the caliper width was 0.2 SDs of the logit of the propensity score.^23^ Covariate balance was assessed between plans using standardized differences.^24,25^

To compare total spending between MA and MSSP, we used generalized estimating equations (GEEs), adjusting for all covariates, with a gamma distribution and log link, and using PCP and zip code fixed effects. We included a PCP fixed effect to account for observable and time-invariant unobservable (eg, practice style) differences in spending across PCPs, which thus represents a site-level characteristic common to all beneficiaries attributed to the same PCP (eg, hours of operation, patient-staff ratio, and ownership status). The model thus only compares outcome differences between MA and MSSP beneficiaries within PCP (ie, attributed to the same PCP). We also included a zip code fixed effect to control for unobserved community features at the level of the zip code of residence for beneficiaries, thus comparing MA and MSSP beneficiaries residing in the same zip code. The GEE adjusted standard errors for the nonindependence of multiple members attributed to the same PCP and for heteroscedasticity. (We used robust standard errors using the Huber-White correction, clustered at the PCP level.)^26,27^ To account for possible changes in coding intensity in the MA cohort, we conducted an additional analysis adjusting for changes in coding patterns between MA and MSSP by creating a regression model to project individual-level Charlson Comorbidity Index for the following year (2016-2018) using demographic, comorbidity, and clinical risk variables from the previous year (2015-2017) and then adjusting for projected Charlson Comorbidity Indices at the beneficiary level for MSSP beneficiaries (as if they had been coded like they were in MA) following prior work.^28^

We tested robustness of our findings to several alternate specifications. First, we used the aggregate Hierarchical Condition Category (HCC) score, which is similar to CMS’s standard risk-adjustment, instead of the Charlson Comorbidity Index. Second, because MSSP beneficiaries who did not have pharmacy claims during 2014 may be systematically different than those who do, we conducted a sensitivity analysis including all beneficiaries with or without a pharmacy claim. Third, it is possible that program differences in pricing between MA and MSSP, most notably the additional provider-based billing charges in MSSP, could contribute to higher MSSP spending. To account for such pricing differences, we identified candidate provider-based billing claims in outpatient and professional claims and deflated MSSP amounts by 20% to observe whether spending differences were sensitive to provider-based billing. Fourth, dual-eligible members may be more likely to switch their MA enrollment and have differential utilization patterns. To account for this, we conducted a sensitivity analysis repeating all analyses excluding dual-eligible members. Fifth, MA beneficiaries may preferentially disenroll from MA near the end of life. To avoid potential bias, we conducted a sensitivity analysis excluding individuals who died in a given year from that year’s analysis. Sixth, high-cost MA beneficiaries may preferentially disenroll from MA during the year. To avoid potential bias caused by disenrolled MA beneficiaries not meeting our continuously enrolled inclusion criterion, we ran a sensitivity analysis using an intent-to-treat approach, following spending over time based on the initial enrollment in MSPP or MA on January 1, 2015.

Analyses were conducted from January 2019 to May 2022. Statistical significance was set at *P* < .05, and all tests were 2-tailed. Analyses were conducted in SAS version 9.4 (SAS Institute).

## Results

The overall sample included 15 763 beneficiaries, 12 720 (81%) in MA and 3043 (19%) in MSSP (eFigure 2 in the Supplement). Our matched comparison group consisted of 11 545 beneficiaries, 4099 in the diabetes cohort, 1354 in the CHF cohort, 3431 in the CKD cohort, and 9727 in the hypertension cohort.

MA and MSSP beneficiaries in each of the 4 disease-specific cohorts differed on a number of characteristics (eTable 4 in the Supplement). For example, MA beneficiaries across all 4 cohorts, compared with MSSP beneficiaries, were more likely to be older (median [IQR] age, 75.0 [69.9-81.8] years vs 73.1 [68.3-79.8] years), male (5515 [43%] vs 1119 [37%]), and White (9644 [76%] vs 2046 [69%]). In contrast, MA beneficiaries were less likely than MSSP beneficiaries to be disabled (827 [7%] vs 297 [10%]), dually eligible (1411 [11%] vs 439 [14%]), and live in low-income zip codes (2338 [19%] vs 750 [25%]). After propensity matching, all observable demographic and clinical characteristics demonstrated standard mean differences of less than 0.12 for the disease-specific cohorts, mostly below the standard of 0.10 for all covariates, indicating adequate match quality (Table 1).^25^

**Table 1.** Baseline Characteristics of MA and MSSP Patients Across Propensity-Score Matched Disease-Specific Cohorts, 2014

Characteristic	Participants, No. (%)											
	Hypertension			Congestive heart failure			Diabetes			Chronic kidney disease		
	MA (n = 7211)	MSSP (n = 2516)	SD	MA (n = 995)	MSSP (n = 359)	SD	MA (n = 3008)	MSSP (n = 1091)	SD	MA (n = 2524)	MSSP (n = 907)	SD
Age, y												
Median (IQR)	73.8 (69.1-80.8)	73.2 (68.5-79.7)	0.11	77.9 (71.2-84.0)	76.9 (70.0-84.0)	0.07	72.9 (68.5-79.8)	72.6 (68.4-78.5)	0.11	75.9 (70.3-81.9)	75.3 (69.3-81.4)	0.10
<50	77 (1.1)	65 (2.6)	0.11	6 (0.6)	7 (1.9)	0.12	27 (0.9)	22 (2.0)	0.09	17 (0.7)	15 (1.7)	0.09
50 to <65	572 (7.9)	226 (9.0)	0.04	80 (8.0)	30 (8.4)	0.01	288 (9.6)	114 (10.4)	0.03	185 (7.3)	77 (8.5)	0.04
65 to <75	3366 (46.7)	1151 (45.7)	0.02	320 (32.2)	116 (32.3)	0.00	1476 (49.1)	527 (48.3)	0.02	972 (38.5)	349 (38.5)	0.00
75 to <85	2326 (32.3)	778 (30.9)	0.03	373 (37.5)	131 (36.5)	0.02	898 (29.9)	321 (29.4)	0.01	1004 (39.8)	340 (37.5)	0.05
≥85	870 (12.1)	296 (11.8)	0.01	216 (21.7)	75 (20.9)	0.02	319 (10.6)	107 (9.8)	0.03	346 (13.7)	126 (13.9)	0.01
Gender												
Male	2760 (38.3)	921 (36.6)	0.04	414 (41.6)	139 (38.7)	0.06	1187 (39.5)	409 (37.5)	0.04	1028 (40.7)	361 (39.8)	0.02
Female	4451 (61.7)	1595 (63.4)	0.04	581 (58.4)	220 (61.3)	0.06	1821 (60.5)	682 (62.5)	0.04	1496 (59.3)	546 (60.2)	0.02
Race												
Black	2073 (28.7)	778 (30.9)	0.05	317 (31.9)	123 (34.3)	0.05	1059 (35.2)	412 (37.8)	0.05	796 (31.5)	318 (35.1)	0.08
Non-Hispanic White	5039 (69.9)	1701 (67.6)	0.05	669 (67.2)	231 (64.3)	0.06	1906 (63.4)	661 (60.6)	0.06	1696 (67.2)	573 (63.2)	0.08
Other^a^	99 (1.4)	37 (1.5)	0.01	9 (0.9)	5 (1.4)	0.05	43 (1.4)	18 (1.6)	0.02	32 (1.3)	16 (1.8)	0.04
Dually eligible	925 (12.8)	360 (14.3)	0.04	225 (22.6)	87 (24.2)	0.04	513 (17.1)	203 (18.6)	0.04	433 (17.2)	178 (19.6)	0.06
Living in a low-income zip code (income <$40 000)	1702 (23.6)	635 (25.2)	0.04	240 (24.1)	91 (25.3)	0.03	770 (25.6)	307 (28.1)	0.06	623 (24.7)	242 (26.7)	0.05
Charlson Comorbidity Index, mean (SD)	2.8 (2.6)	2.9 (2.6)	0.02	5.6 (2.8)	5.4 (2.6)	0.05	4.4 (2.7)	4.3 (2.6)	0.04	5.1 (2.4)	5.0 (2.3)	0.05
BMI												
Mean (SD)	29.6 (6.4)	29.8 (6.7)	0.04	30.1 (6.9)	30.4 (7.3)	0.05	31.4 (6.5)	31.7 (6.9)	0.05	29.9 (6.4)	29.8 (6.6)	0.02
<18.5	107 (1.5)	39 (1.6)	0.00	26 (2.6)	9 (2.5)	0.01	19 (0.6)	7 (0.6)	0.01	45 (1.8)	18 (2.0)	0.01
18.5 to <25	1746 (24.2)	584 (23.2)	0.02	212 (21.3)	77 (21.4)	0.00	455 (15.1)	161 (14.8)	0.01	511 (20.2)	195 (21.5)	0.03
25 to <30	2366 (32.8)	814 (32.4)	0.01	297 (29.8)	102 (28.4)	0.03	901 (30.0)	327 (30.0)	0.00	869 (34.4)	306 (33.7)	0.01
30 to <40	2446 (33.9)	880 (35.0)	0.02	377 (37.9)	138 (38.4)	0.01	1305 (43.4)	465 (42.6)	0.02	902 (35.7)	317 (35.0)	0.02
≥40	546 (7.6)	199 (7.9)	0.01	83 (8.3)	33 (9.2)	0.03	328 (10.9)	131 (12.0)	0.03	197 (7.8)	71 (7.8)	0.00
Smoking status												
Current	493 (6.8)	186 (7.4)	0.02	53 (5.3)	21 (5.8)	0.02	206 (6.8)	76 (7.0)	0.01	141 (5.6)	54 (6.0)	0.02
Former	2826 (39.2)	967 (38.4)	0.02	467 (46.9)	155 (43.2)	0.07	1238 (41.2)	443 (40.6)	0.01	1033 (40.9)	359 (39.6)	0.03
Never	3892 (54.0)	1363 (54.2)	0.00	475 (47.7)	183 (51.0)	0.07	1564 (52.0)	572 (52.4)	0.01	1350 (53.5)	494 (54.5)	0.02
Systolic blood pressure, mm Hg												
Mean (SD)	135.8 (13.9)	135.3 (13.5)	0.04	133.4 (14.9)	133.5 (14.3)	0.00	135.9 (13.8)	135.4 (13.7)	0.03	135.4 (13.9)	135.1 (14.0)	0.02
<120	816 (11.3)	303 (12.0)	0.02	190 (19.1)	68 (18.9)	0.00	327 (10.9)	128 (11.7)	0.03	312 (12.4)	120 (13.2)	0.02
120 to <140	3860 (53.5)	1348 (53.6)	0.00	488 (49.0)	178 (49.6)	0.01	1617 (53.8)	590 (54.1)	0.01	1354 (53.6)	482 (53.1)	0.01
140 to ≤180	2502 (34.7)	853 (33.9)	0.02	315 (31.7)	112 (31.2)	0.01	1050 (34.9)	367 (33.6)	0.03	845 (33.5)	299 (33.0)	0.01
>180	33 (0.5)	12 (0.5)	0.00	2 (0.2)	1 (0.3)	0.02	14 (0.5)	6 (0.5)	0.01	13 (0.5)	6 (0.7)	0.02
Creatinine level, mg/dL												
Mean (SD)	1.1 (0.3)	1.1 (0.3)	0.02	1.2 (0.5)	1.2 (0.5)	0.07	1.1 (0.4)	1.1 (0.4)	0.03	1.3 (0.5)	1.3 (0.5)	0.00
0.6 to <1.2	5217 (72.3)	1826 (72.6)	0.01	571 (57.4)	209 (58.2)	0.02	1987 (66.1)	722 (66.2)	0.00	1162 (46.0)	417 (46.0)	0.00
1.2 to ≤2.0	1861 (25.8)	640 (25.4)	0.01	360 (36.2)	132 (36.8)	0.01	938 (31.2)	341 (31.3)	0.00	1223 (48.5)	437 (48.2)	0.01
>2.0	133 (1.8)	50 (2.0)	0.01	64 (6.4)	18 (5.0)	0.06	83 (2.8)	28 (2.6)	0.01	139 (5.5)	53 (5.8)	0.01
ACE inhibitors	5048 (70.0)	1747 (69.4)	0.01	709 (71.3)	257 (71.6)	0.01	2235 (74.3)	799 (73.2)	0.02	1725 (68.3)	615 (67.8)	0.01
HbA_1c_ level	6.8 (1.2)	6.9 (1.2)	0.09									
Mean (SD), %	6.8 (1.2)	6.9 (1.2)	0.09	NA	NA	NA	7.0 (1.2)	7.0 (1.2)	0.03	7.0 (1.2)	7.0 (1.3)	0.07
<6%	NA	NA	NA	NA	NA	NA	416 (13.8)	147 (13.5)	0.01	NA	NA	NA
6 to <7%	NA	NA	NA	NA	NA	NA	1381 (45.9)	499 (45.7)	0.00	NA	NA	NA
7 to ≤8%	NA	NA	NA	NA	NA	NA	762 (25.3)	276 (25.3)	0.00	NA	NA	NA
>8%	NA	NA	NA	NA	NA	NA	449 (14.9)	169 (15.5)	0.02	NA	NA	NA
Charlson comorbidities												
Myocardial infarction	401 (5.6)	156 (6.2)	0.03	NA	NA	NA	211 (7.0)	71 (6.5)	0.02	NA	NA	NA
Insulin	NA	NA	NA	NA	NA	NA	762 (25.3)	292 (26.8)	0.03	NA	NA	NA
Statins	NA	NA	NA	NA	NA	NA	2246 (74.7)	802 (73.5)	0.03	NA	NA	NA
Diabetes	NA	NA	NA	512 (51.5)	179 (49.9)	0.03	NA	NA	NA	1390 (55.1)	501 (55.2)	0.00
Chronic pulmonary disease	NA	NA	NA	277 (27.8)	95 (26.5)	0.03	NA	NA	NA	NA	NA	NA
β blockers	NA	NA	NA	812 (81.6)	287 (79.9)	0.04	NA	NA	NA	NA	NA	NA
Diuretics	NA	NA	NA	740 (74.4)	293 (81.6)	0.17	NA	NA	NA	NA	NA	NA
Ejection fraction												
Mean (SD)	NA	NA	NA	49.3 (14.5)	49.0 (14.8)	0.02	NA	NA	NA	NA	NA	NA
<35	NA	NA	NA	83 (8.3)	35 (9.7)	0.05	NA	NA	NA	NA	NA	NA
35 to <45	NA	NA	NA	52 (5.2)	19 (5.3)	0.00	NA	NA	NA	NA	NA	NA
45 to ≤55	NA	NA	NA	157 (15.8)	58 (16.2)	0.01	NA	NA	NA	NA	NA	NA
>55	NA	NA	NA	178 (17.9)	67 (18.7)	0.02	NA	NA	NA	NA	NA	NA
Missing	NA	NA	NA	525 (52.8)	180 (50.1)	0.05	NA	NA	NA	NA	NA	NA

Abbreviations: ACE, angiotensin-converting enzyme; BMI, body mass index (calculated as weight in kilograms divided by height in meters squared); HbA_1c_, hemoglobin A_1c_; MA, Medicare Advantage; MSSP, Medicare Shared Savings Program; NA, not applicable.

SI conversion factor: To convert creatinine to micromoles per liter, multiply by 88.4; HbA_1c_ to proportion of total hemoglobin, multiply by 0.01.

^a^ Other race includes Asian, American Indian or Alaskan Native, Hispanic or Latino, Native Hawaiian or Pacific Islander, other, unknown or missing, and patient refused to provide.

### Spending Differences Between MSSP and MA Across Disease Cohorts

For disease-specific cohorts over the study follow-up period, mean unadjusted per-member per-year (PMPY) spending differences between MSSP and MA were $2159 (diabetes), $4074 (CHF), $2560 (CKD), and $2335 (hypertension). After propensity score matching and including zip code and PCP fixed effects, PMPY spending differences between MSSP and MA decreased from $2434 in 2015 to $2280 in 2018 in the diabetes cohort and from $4740 to $2960 in the CHF cohort; but increased from $2628 to $3005 in the CKD cohort and $1895 to $2115 in the hypertension cohort (Table 2). Adjusted MSSP spending remained 23% to 30% higher than MA spending across the follow-up period in all disease cohorts (Figure). Outpatient hospital spending contributed most to higher MSSP overall spending (mean outpatient hospital spending difference relative to MA: $1581 in diabetes; $2014 in CHF; $1695 in CKD; $1456 in hypertension) (Table 3). Primary care spending was significantly lower for MSSP in the diabetes (mean primary care spending difference relative to MA: −$98), CKD (−$87), and hypertension (−$108) cohorts but not the CHF cohort (−$14). Inpatient spending was significantly higher for MSSP in all disease cohorts (mean inpatient spending difference relative to MA: $585 in diabetes; $1874 in CHF; $897 in CKD; $613 in hypertension). Non–primary care specialist spending was not significantly different between MSSP and MA beneficiaries across all disease cohorts. Utilization differences were consistent with spending differences.

**Table 2.** Unadjusted and Adjusted Differences in Overall Spending in 2015

Adjustment	Absolute difference (95% CI), $			
	Hypertension	CHF	Diabetes	CKD
Unadjusted^a^	2416 (1832-3032)	3605 (1426-6058)	2456 (1575-3408)	2603 (1539-3755)
Adjusting				
For demographic characteristics and comorbidities^b^	2276 (1653-2937)	4483 (2080-7207)	2829 (1835-3909)	2493 (1324-3772)
For relevant claims-based characteristics^c^	2273 (1634-2953)	5194 (2610-8141)	3076 (2043-4201)	2681 (1433-4053)
For relevant claims and clinical characteristics^d^	2202 (1535-2914)	4872 (2257-7864)	2990 (1870-4220)	2752 (1429-4214)
For relevant claims, clinical characteristics, and SES^e^	2183 (1504-2908)	4917 (2303-7909)	2888 (1759-4129)	2849 (1506-4335)
For relevant claims, clinical characteristics, and SES, zip code and PCP fixed effects^f^	2113 (1476-2791)	4470 (1874-7447)	2835 (1832-3938)	2793 (1598-4100)
Propensity score				
Matched^g^	2258 (1616-2939)	3699 (1235-6523)	2454 (1431-3574)	2478 (1172-3920)
Matched and coding adjusted	2009 (1338-2726)	4624 (1789-7921)	2719 (1591-3964)	2874 (1424-4489)
Matched and adjusted for relevant claims, clinical characteristics, and SES, zip code and PCP fixed effects^f^	1895 (1261-2569)	4740 (1764-8227)	2434 (1478-3475)	2628 (1380-3999)

Abbreviations: CHF, congestive heart failure; CKD, chronic kidney disease; MA, Medicare Advantage; MSSP, Medicare Shared Savings Program; PCP, primary care physician; SES, socioeconomic status.

^a^ The mean (SD) spending for the hypertension cohort was $8556 ($14 208) for MA and $10 971 ($16 286) for MSSP; for CHF cohort, it was $15 832 ($19 923) for MA and $19 347 ($20 479) for MSSP; for diabetes, it was $9515 ($15 009) for MA and $11 972 ($17 353) for MSSP; for CKD, it was $11 125 ($16 885) for MA and $13 727 ($17 737) for MSSP.

^b^ Adjusted for age, sex, race, and Charlson Comorbdity Index.

^c^ Adjusted for age, sex, race, Charlson Comorbidity Index, dual eligibility, and cohort-specific comorbidities (myocardial infarction in hypertension and diabetes cohorts; diabetes in CHF and CKD cohorts; and chronic obstructive pulmonary disease in the CHF cohort).

^d^ Adjusted for age, sex, race, Charlson Comorbidity Index, dual eligibility, cohort-specific comorbidities, and cohort-specific medications (insulins and statins in diabetes cohort; β-blockers in CHF cohort; angiotensin-converting enzyme inhibitors in all cohorts), body mass index, smoking, creatinine level, and systolic blood pressure as well as hemoglobin A_1c_ level in the hypertension, diabetes, and CKD cohorts and ejection fraction in the CHF cohort.

^e^ Adjusted for age, sex, race, Charlson Comorbidity Index, dual eligibility, cohort-specific comorbidities, cohort-specific medications, body mass index, smoking, creatinine level, and systolic blood pressure, and area-level socioeconomic characteristics as well as hemoglobin A_1c_ level in the hypertension, diabetes, and CKD cohorts and ejection fraction in the CHF cohort.

^f^ Adjusted for age, sex, race, Charlson Comorbidity Index, dual eligibility, cohort-specific comorbidities, cohort-specific medications, smoking, creatinine level, systolic blood pressure, area-level socioeconomic characteristics, and PCP and zip code fixed effects as well as hemoglobin A_1c_ level in the hypertension, diabetes, and CKD cohorts and ejection fraction in the CHF cohort.

^g^ The mean (SD) spending for the propensity-matched hypertension cohort was $8637 ($14 290) for MA and $10 895 ($16 329) for MSSP; for CHF cohort, it was $15 606 ($19 468) for MA and $19 305 ($20 697) for MSSP; for diabetes, it was $9297 ($14 694) for MA and $11 751 ($17 125) for MSSP; for CKD, it was $11 324 ($17 205) for MA and $13 802 ($18 041) for MSSP.

**Figure.**
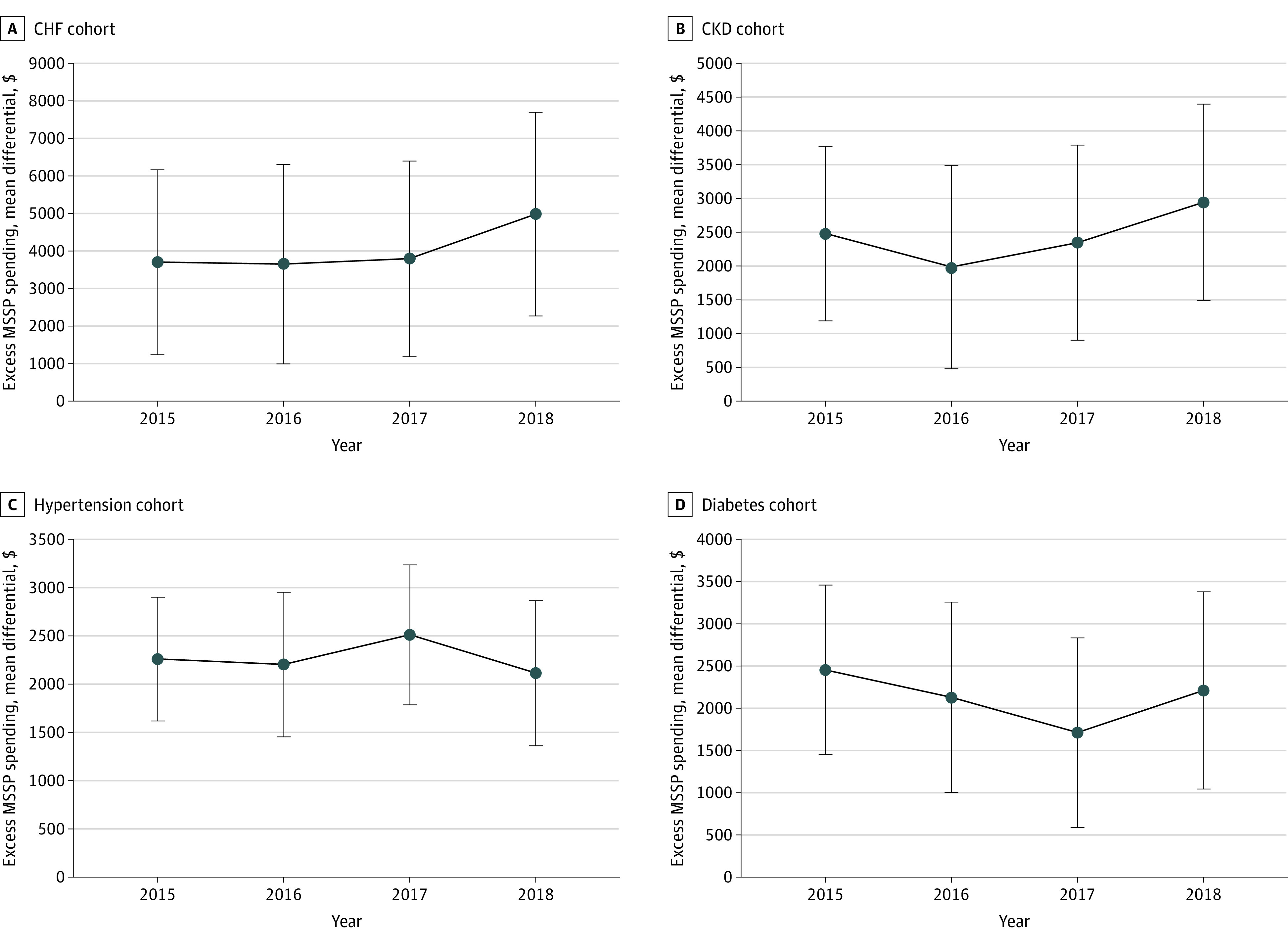
Changes in Differential Spending Over Time in Medicare Advantage and Medicare Shared Savings Program (MSSP) Cohorts Trends in overall spending differences between propensity-score matched Medicare Advantage and MSSP cohorts. Bars indicate 95% CIs. CHF indicates congestive heart failure; and CKD, chronic kidney disease.

**Table 3.** Differences in Spending for Propensity-Matched Medicare Advantage and Medicare Shared Savings Program Cohorts Over Time, 2015-2018

Category	Difference, spending per member, $^a^							
	CHF		CKD		Diabetes		Hypertension	
	2015	2018	2015	2018	2015	2018	2015	2018
Total cost of care	3699^b^	4979^c^	2478^c^	2942^b^	2454^b^	2209^c^	2258^b^	2113^b^
Primary care	−42	−14	−144^b^	−87^c^	−149^b^	−98^b^	−156^b^	−108^b^
Hospital								
Inpatient	458	1874^b^	199	897^b^	282	585^d^	304	613^c^
Outpatient	2014^b^	1715^c^	1695^b^	1472^b^	1581^b^	1291^b^	1456^b^	1143^b^
Emergency department	−54	−64	−5	−106^b^	−10	−64^d^	−2	−20
Other non–primary care professional	−175	113	−158	−2	−91	−97	−70	20

Abbreviations: CHF, congestive heart failure; CKD, chronic kidney disease.

^a^ All differences are calculated as Medicare Shared Savings Program spending minus Medicare Advantage spending.

^b^ *P* < .01.

^c^ *P* < .001.

^d^ *P* < .05.

### Sensitivity Analyses

Sensitivity analyses that used HCCs for comorbidity adjustment (eTable 5 in the Supplement), included beneficiaries without a pharmacy claim (eTable 6 in the Supplement), accounted for provider-based billing (eFigure 3 in the Supplement), excluded dual-eligible members (eFigure 4 in the Supplement), excluded decedents (eFigure 5 in the Supplement), or included noncontinuously enrolled individuals in an intent-to-treat approach (eFigure 6 in the Supplement) did not meaningfully change results.

## Discussion

We found that average PMPY spending was 22% to 26% higher for MSSP ACO beneficiaries than for MA beneficiaries even after controlling for clinical risk and many other factors. Across all 4 disease-specific cohorts, this was largely associated with higher outpatient hospital and inpatient spending for MSSP beneficiaries. In this health system, practice patterns and quality of care were similar for MA and MSSP, which are subject to different mechanisms of spending control (global capitation and shared savings, respectively). Despite this, accounting for clinical and area-level socioeconomic factors and differences in coding intensity did not explain the greater spending for MSSP beneficiaries. This study has 2 important policy implications for policy makers and participating health systems with respect to designing programs that attempt to control costs for Medicare beneficiaries.

First, after adjusting for clinical risk, per-beneficiary spending for MSSP was higher than for MA, and the gap slightly widened for most cohorts over the study period. These findings are unlikely to be primarily explained by clinician, health system, or practice pattern factors, given our use of clinician and zip code fixed effects and our study of a single health system with similar practice patterns and quality of care for MA and MSSP beneficiaries. Additionally, differences persisted after standardizing prices, arguing that lower negotiated reimbursement rates for MA beneficiaries do not explain our findings.

What drives spending differences is likely multifactorial. While controlling for area-level socioeconomic factors did not affect spending differences, it is likely that unmeasured selection factors relating to more adverse socioeconomic factors for MSSP beneficiaries may contribute to higher MSSP spending. These may include unstable housing or transportation that predispose to higher acute care utilization. Additionally, payer-specific levers, such as utilization management, may contribute. Payers, generally not health systems, use utilization management to control spending for MA beneficiaries. Utilization management policies in MA would apply to a large proportion of FFS beneficiaries but are not allowed in Medicare FFS.^29^ Finally, differences in MA and MSSP program design may contribute. Variability in factors such as site of service and differences in plan design, notably additional levers for MA to control costs through network design, may further contribute to lower outpatient hospital spend among MA beneficiaries.^16^

Second, adding the EHR clinical data to standard claims-based variables accounted for only a small amount of observed spending differences between MA and MSSP beneficiaries. This suggests that the effort and cost of adding EHR clinical data to large scale risk-adjustment models such as the CMS-HCC score may not be justified. This is one of the first studies using detailed EHR data to account for differences in clinical risk between MA and MSSP. Over the past 20 years, CMS has undertaken several efforts to improve risk adjustment in the MA program to account for clinical and socioeconomic differences between MA and FFS populations.^14,15,29^ These have included expanding diagnostic information to include both inpatient and outpatient information under the CMS-HCC and better accounting for specific factors such as substance use, mental health, and CKD. Our study expands on these improvements in risk adjustment by incorporating rich EHR-based clinical information to estimate risk using clinically validated risk measures derived from clinical practice. Nonclinical risk factors and factors related to plan design likely play an outsized role in unexplained spending differences between the MA and FFS populations. Collecting individual social determinants of health or other data on health behaviors and individual characteristics (eg, health literacy, engagement in personal health) may be important for accurate risk adjustment.

Health systems may be less likely to achieve spending targets through the MSSP program compared with the MA program, despite providing similar high-quality care regardless of its beneficiaries’ program enrollment. One reason for this could relate to program design. For example, to assign patients to clinicians, MSSP requires use of primary care services, which at the time of this study included postacute care. Such requirements were not present for MA. Thus, MSSP beneficiaries assigned to OHS may have had mechanically higher spending than other FFS beneficiaries because they exclude those without any qualifying services for the year. For program design-related reasons such as this, a health system that cares for both MA and MSSP beneficiaries may be disadvantaged for each MSSP beneficiary it takes on. While there could be some residual patient or geographic factors that influence the spending difference, based on our analysis it is unlikely that these factors would account for the full difference observed. As unmeasured socioeconomic factors may account for this residual spending difference, our analysis highlights the need for more reliable collection of data that reflect socioeconomic status, social factors, and social determinants of health.

Our findings provide a programmatic perspective with regards to health system participation in MSSP vs MA. We suggest that health systems that take on risk for FFS beneficiaries through programs like MSSP may face a disadvantage compared with those that primarily take on risk for MA, despite using similar types of care redesign efforts and common infrastructure. This may create a problem for CMS to achieve its goal of garnering broad clinician and health system participation in ACO programs as a means to achieve 100% FFS beneficiary alignment with alternative payment models.^30^ Changes to program design to increase symmetry may be required. For example, direct contracting arrangements could offer health systems and clinicians participating in MSSP and other risk-bearing FFS arrangements more flexibility to manage spending. MA and MSSP program design should also reflect the inherent differences in beneficiaries. More generous MSSP spending targets could account for potential adverse selection effects that disadvantage MSSP. Such generous MSSP targets could allow financial benchmarks to be more aligned between MSSP and MA beneficiaries, so that success in one program translates to success in the other. This may incentivize health systems to participate and take on risk for MSSP, rather than being disincentivized from participating in MSSP in favor of routing select patients toward MA. Another path could be to directly adjust for observable area-level social determinant of health measures, which the Medicare ACO REACH program is intending to do. However, our study suggests that this may be an incomplete offset. The need for better symmetry in program design between MA and FFS Medicare has been recognized by many organizations including the Medicare Payment Advisory Commission.^28^

### Limitations

This study had several limitations. First, it was an observational study, and thus our results could be confounded by omitted variables and unobservable differences between beneficiaries and the clinicians who care for them, such as socioeconomic status. Such beneficiaries may also cluster among a select group of PCPs, which may also influence outcomes. Additionally, it was difficult to account for unobserved selection effects that may have routed certain patients into the MA program or resulted in differential rates of disenrollment, including differential disenrollment from MA at the end of life. We conducted several sensitivity analyses suggesting that such factors did not likely drive our results, and we used several techniques to mitigate such confounding, including using clinically validated risk algorithms to identify beneficiaries of similar risk and using propensity matching. We also only used comparisons within PCP and zip code to account for unobserved characteristics that may influence outcomes, although these variables may not account for all socioeconomic characteristics which would otherwise influence utilization and there still may be socioeconomic heterogeneity within zip codes and PCP panels. Second, we only examined comparisons between MA and MSSP beneficiaries, rather than all Medicare FFS beneficiaries seen at OHS, as we wished to only compare beneficiaries for whom OHS and physicians assumed risk for total cost of care. Third, our results study a single health system within the southern United States that may not generalize to other MA and FFS populations. However, no national EHR exists that would allow a nationwide study accounting for the same variables we consider; thus, this is likely a common issue in any studies. Furthermore, our cohort received care within several practices within a large health system serving a racially and ethnically diverse patient population, and thus likely carries relevance for a large proportion of the United States. Fourth, upcoding within one of the programs could also affect who gets included in the condition-specific cohorts. However, we risk-adjusted using clinical metrics (such as baseline creatinine and hemoglobin A_1c_ levels) that should not be subject to upcoding and thus should mitigate this concern. Fifth, factors other than residual differences in underlying populations between MA and MSSP may also contribute to our findings. However, our findings were robust even after standardizing for provider-based billing differences, suggesting that price standardization is unlikely to ameliorate underlying spending differences. Sixth, our GEE approach used robust standard errors using the Huber-White correction at the PCP level and assumes homoskedasticity across PCPs, which, if untrue, may result in inaccurate standard errors.

## Conclusions

In conclusion, using administrative and electronic health record data from a large academic health system, we found that utilization and spending patterns vary considerably between MSSP and MA beneficiaries, even when controlling for rich metrics of clinical risk. Health system participation in MA may be more favorable than MSSP because of an inability to manage nonclinical risk factors; changes to program financial design and better accounting for unmeasured covariates may help to mitigate this disparity.

## Notes

Supplement. **eTable 1. **Comparison of Quality Metrics between MA and MSSP Members **eTable 2. ***ICD-9* and *ICD-10* Codes Included in Cohort Definitions **eTable 3. **Covariates Included in Propensity Matching Models Across 4 Disease Cohorts **eTable 4. **Baseline Characteristics of MA and MSSP Patients before Propensity Score Matching, 2014 **eTable 5. **Unadjusted and Adjusted Differences in Overall Spending Using HCCs Instead of Charlson Comorbidities **eTable 6. **Unadjusted and Adjusted Differences in Overall Spending (Charlson Comorbidities) Including MSSP Beneficiaries Without a Pharmacy Claim **eFigure 1. **Practice Patterns among MSSP and MA Beneficiaries **eFigure 2. **Cohort Selection **eFigure 3. **Unadjusted and Adjusted Overall Spending in MSSP After Adjusting for Provider-Based Billing **eFigure 4. **Sensitivity Analysis of Changes in Differential Spending Over Time in MA and MSSP Cohorts, Excluding Decedents **eFigure 5. **Sensitivity Analysis of Changes in Differential Spending Over Time in MA and MSSP Cohorts, Excluding Dual-Eligible Beneficiaries **eFigure 6. **Sensitivity Analysis of Changes in Differential Spending Over Time in MA and MSSP Cohorts, Using Intent-to-Treat Approach
