# Supplementary material for: Evaluation of Spending Differences Between Beneficiaries in Medicare Advantage and the Medicare Shared Savings Program
Source: JAMA Netw Open. 2022 Aug 23;5(8):e2228529. doi: 10.1001/jamanetworkopen.2022.28529 (PMC9399862; doi:10.1001/jamanetworkopen.2022.28529)
Supplement: Supplement. — eTable 1. Comparison of Quality Metrics between MA and MSSP Members eTable 2. ICD-9 and ICD-10 Codes Included in Cohort Definitions eTable 3. Covariates Included in Propensity Matching Models Across 4 Disease Cohorts eTable 4. Baseline Characteristics of MA and MSSP Patients before Propensity Score Matching, 2014 eTable 5. Unadjusted and Adjusted Differences in Overall Spending Using HCCs Instead of Charlson Comorbidities eTable 6. Unadjusted and Adjusted Differences in Overall Spending (Charlson Comorbidities) Including MSSP Beneficiaries Without a Pharmacy Claim eFigure 1. Practice Patterns among MSSP and MA Beneficiaries eFigure 2. Cohort Selection eFigure 3. Unadjusted and Adjusted Overall Spending in MSSP After Adjusting for Provider-Based Billing eFigure 4. Sensitivity Analysis of Changes in Differential Spending Over Time in MA and MSSP Cohorts, Excluding Decedents eFigure 5. Sensitivity Analysis of Changes in Differential Spending Over Time in MA and MSSP Cohorts, Excluding Dual-Eligible Beneficiaries eFigure 6. Sensitivity Analysis of Changes in Differential Spending Over Time in MA and MSSP Cohorts, Using Intent-to-Treat Approach [file jamanetwopen-e2228529-s001.pdf]

Supplemental Online Content

Parikh RB, Emanuel EJ, Brensinger CM, et al. Evaluation of spending differences between beneficiaries in Medicare Advantage and the Medicare Shared Savings Program. *JAMA Netw Open*. 2022;5(8):e2228529. doi:10.1001/jamanetworkopen.2022.28529

eTable 1. Comparison of Quality Metrics between MA and MSSP Members

eTable 2. *ICD-9* and *ICD-10* Codes Included in Cohort Definitions

eTable 3. Covariates Included in Propensity Matching Models Across 4 Disease Cohorts

eTable 4. Baseline Characteristics of MA and MSSP Patients before Propensity Score Matching, 2014

eTable 5. Unadjusted and Adjusted Differences in Overall Spending Using HCCs Instead of Charlson Comorbidities

eTable 6. Unadjusted and Adjusted Differences in Overall Spending (Charlson Comorbidities) Including MSSP Beneficiaries Without a Pharmacy Claim

eFigure 1. Practice Patterns among MSSP and MA Beneficiaries

eFigure 2. Cohort Selection

eFigure 3. Unadjusted and Adjusted Overall Spending in MSSP After Adjusting for Provider-Based Billing

eFigure 4. Sensitivity Analysis of Changes in Differential Spending Over Time in MA and MSSP Cohorts, Excluding Decedents

eFigure 5. Sensitivity Analysis of Changes in Differential Spending Over Time in MA and MSSP Cohorts, Excluding Dual-Eligible Beneficiaries

eFigure 6. Sensitivity Analysis of Changes in Differential Spending Over Time in MA and MSSP Cohorts, Using Intent-to-Treat Approach

This supplemental material has been provided by the authors to give readers additional information about their work.

© 2022 Parikh RB et al. *JAMA Network Open.*

eTable 1. Comparison of Quality Metrics between MA and MSSP members

	Hypertension				Diabetes				Congestive Heart Failure				Chronic Kidney Disease			
Measure	MA[a]	MSSP[b]	Diff	P Value	MA	MSSP	Diff	P Value	MA	MSSP	Diff	P Value	MA	MSSP	Diff	P Value
Controlling High Blood Pressure	73.7	71.9	1.8	0.15	72.7	70.5	2.2	0.28	74.3	76.2	-1.9	0.65	71.7	69.9	1.8	0.45
Statin Therapy for the Prevention and Treatment of Cardiovascular Disease	79.4	78.6	0.7	0.53	87.6	87.0	0.6	0.69	83.5	84.4	-1.0	0.75	**81.7**	**77.3**	**4.4**	**0.02**
Ischemic Vascular Disease (IVD): Use of Aspirin or Another Antiplatelet	**63.2**	**68.4**	**-5.3**	**0.01**	68.4	69.8	-1.4	0.64	75.8	70.8	5.0	0.40	66.2	69.5	-3.3	0.35
Annual Visit with a Primary Care Provider	98.9	98.8	0.1	0.74	98.6	98.9	-0.3	0.50	98.8	98.6	0.2	0.80	99.0	99.7	-0.7	0.07
Comprehensive Diabetes Care (CDC) - Eye exam (retinal) performed	**78.6**	**88.3**	**-9.7**	**0.00**	**78.5**	**89.4**	**-10.9**	**0.00**	75.3	72.2	3.1	0.79	**77.9**	**87.7**	**-9.8**	**0.02**
(CDC) - HbA1c Poor Control (greater than 9%)	10.6	11.2	-0.5	0.78	9.9	10.3	-0.4	0.84	18.5	5.6	13.0	0.18	13.2	9.0	4.2	0.23

© 2022 Parikh RB et al. *JAMA Network Open.*

(CDC) - HbA1c Testing	96.5	95.4	1.1	0.36	96.7	96.0	0.7	0.54	97.5	100.0	-2.5	0.50	96.3	95.9	0.4	0.84
(CDC) - HbA1c control (less than 8.0%)	81.0	81.2	-0.2	0.92	81.7	81.7	0.1	0.98	75.3	83.3	-8.0	0.47	79.4	83.6	-4.2	0.32
(CDC) - Medical Attention for Nephropathy	**98.1**	**95.4**	**2.7**	**0.01**	97.8	96.0	1.8	0.07	98.8	100.0	-1.2	0.64	99.1	98.4	0.7	0.52
Controlling High Blood Pressure (CBP) - version 2	76.3	73.7	2.5	0.07	**76.1**	**71.7**	**4.4**	**0.05**	75.8	80.6	-4.8	0.42	77.0	72.5	4.5	0.10

[a]MA = Medicare Advantage
[b]MSSP = Medicare Shared Savings Program

© 2022 Parikh RB et al. *JAMA Network Open.*

eTable 2. ICD-9 and ICD-10 Codes Included in Cohort Definitions

	Hypertension	Congestive Heart Failure	Diabetes	Chronic Kidney Disease
**ICD-9[a]**	401.x, 402.x, 403.x, 404.x, 405.x	398.91, 402.01, 402.11, 402.91, 404.01, 404.03, 404.11, 404.13, 404.91, 404.93, 425.4–425.9, 428.x	250.0–250.3, 250.8, 250.9, 250.4–250.7	403.01, 403.11, 403.91, 404.02, 404.03, 404.12, 404.13, 404.92, 404.93, 582.x, 583.0–583.7, 585.x, 586.x, 588.0, V42.0, V45.1, V56.x
**ICD-10**	I10.x, I11.x, I12.x, I15.x	I09.9, I11.0, I13.0, I13.2, I25.5, I42.0, I42.5–I42.9, I43.x, I50.x, P29.0	E10.0, E10.1, E10.6, E10.8, E10.9, E11.0, E11.1, E11.6, E11.8, E11.9, E12.0, E12.1, E12.6, E12.8, E12.9, E13.0, E13.1, E13.6, E13.8, E13.9, E14.0, E14.1, E14.6, E14.8, E14.9, E10.2–E10.5, E10.7, E11.2–E11.5, E11.7, E12.2–E12.5, E12.7, E13.2–E13.5, E13.7, E14.2–E14.5, E14.7	I12.0, I13.1, N03.2–N03.7, N05.2–N05.7, N18.x, N19.x, N25.0, Z49.0–Z49.2, Z94.0, Z99.2
[a]ICD = International Statistical Classification of Diseases and Related Health Problems				

© 2022 Parikh RB et al. *JAMA Network Open.*

eTable 3. Covariates Included in Propensity Matching Models Across 4 Disease Cohorts

**Hypertension**	**Congestive Heart Failure**	**Diabetes**	**Chronic Kidney Disease**	**Notes**
Age Group	Age Group	Age Group	Age Group	<50, 50 To <65, 65 To <75, 75 To <85, 85+
Race	Race	Race	Race	White, African American, Other
Gender	Gender	Gender	Gender	
Charlson Index	Charlson Index	Charlson Index	Charlson Index	
BMI[a]	BMI	BMI	BMI	<18.5, 18.5 To <25, 25 To <30, 30 To <40, 40+
Smoking	Smoking	Smoking	Smoking	Current, Former, Never
Systolic BP[b]	Systolic BP	Systolic BP	Systolic BP	<120, 120 To <140, 140 To ≤ 180, >180
Creatinine	Creatinine	Creatinine	Creatinine	0.6 To <1.2, 1.2 To ≤ 2, >2
	Ejection Fraction			<35, 35 To <45, 45 To ≤ 55, >55
		Hba1c[d]		<6, 6 To <7, 7 To ≤8, >8
MI[c]		MI		
	Diabetes		Diabetes	
	COPD			
Ace Inhibitors	Ace Inhibitors	Ace Inhibitors	Ace Inhibitors	
	Beta Blockers			
		Insulin		
		Statins		
Low Income Zip Code	Low Income Zip Code	Low Income Zip Code	Low Income Zip Code	<$40k
Dual Eligibility	Dual Eligibility	Dual Eligibility	Dual Eligibility	
[a]BMI = body mass index<br>[b]BP = blood pressure<br>[c]MI = myocardial				

© 2022 Parikh RB et al. *JAMA Network Open.*

infarction<br>[d]Hba1c = hemoglobin A1C

© 2022 Parikh RB et al. *JAMA Network Open.*

eTable 4. Baseline Characteristics of MA and MSSP Patients before Propensity Score Matching, 2014(continued on next page)

	Hypertension			Congestive Heart Failure			Diabetes			Chronic Kidney Disease		
	MA[a]	MSSP[b]	SD[c]	MA	MSSP	SD	MA	MSSP	SD	MA	MSSP	SD
**Median age, y (IQR)[d]**	75.1 (70.0-81.9)	73.2 (68.4-79.9)	0.24	79.3 (72.2-85.0)	77.2 (69.8-84.0)	0.20	74.3 (69.3-80.8)	72.5 (68.2-78.9)	0.26	77.0 (71.1-83.4)	75.4 (69.2-81.7)	0.24
**Gender**												
**Male**	5,170 (43.0%)	1,039 (36.2%)	0.14	859 (47.6%)	151 (37.8%)	0.20	2,363 (47.5%)	516 (38.3%)	0.19	2,167 (47.0%)	407 (39.6%)	0.15
**Female**	6,850 (57.0%)	1,828 (63.8%)	0.14	946 (52.4%)	249 (62.3%)	0.20	2,612 (52.5%)	831 (61.7%)	0.19	2,446 (53.0%)	622 (60.4%)	0.15
**Race**												
**non-Hispanic White**	9,039 (75.5%)	1,903 (67.7%)	0.17	1,330 (74.0%)	252 (64.3%)	0.21	3,437 (69.3%)	811 (61.1%)	0.17	3,421 (74.4%)	641 (63.9%)	0.23
**Black**	2,806 (23.4%)	870 (30.9%)	0.17	459 (25.5%)	135 (34.4%)	0.20	1,467 (29.6%)	496 (37.3%)	0.16	1,134 (24.7%)	346 (34.5%)	0.22
**Other**	125 (1.0%)	39 (1.4%)	0.04	9 (0.5%)	5 (1.3%)	0.08	59 (1.2%)	21 (1.6%)	0.03	42 (0.9%)	16 (1.6%)	0.06
**Current entitlement reason Disabled (vs. Aged)**	751 (6.3%)	261 (9.1%)	0.11	108 (6.0%)	32 (8.0%)	0.08	403 (8.1%)	147 (10.9%)	0.10	244 (5.3%)	92 (8.9%)	0.14
**Dually eligible**	1,347 (11.2%)	419 (14.6%)	0.10	290 (16.1%)	102 (25.5%)	0.23	701 (14.1%)	249 (18.5%)	0.12	555 (12.0%)	199 (19.3%)	0.20
**% living in a low-income zip code (income <$40,000)**	2,221 (18.8%)	713 (25.5%)	0.16	356 (20.0%)	99 (25.2%)	0.12	1,030 (21.1%)	362 (27.7%)	0.15	923 (20.3%)	266 (26.5%)	0.15
**% living in a low education zip code (% with high school education <80%)**	2,374 (20.1%)	594 (21.2%)	0.03	349 (19.6%)	112 (28.5%)	0.21	1,131 (23.1%)	305 (23.2%)	0.00	927 (20.3%)	237 (23.6%)	0.08
**Mean Charlson Comorbidity Index**	2.7 (2.6)	2.8 (2.6)	0.04	5.5 (2.7)	5.4 (2.6)	0.01	4.4 (2.6)	4.3 (2.6)	0.06	4.9 (2.4)	5.0 (2.3)	0.03
**Mean BMI**	29.3 (6.0)	29.9 (6.7)	0.10	29.8 (6.8)	30.5 (7.3)	0.10	30.8 (6.2)	31.6 (6.9)	0.13	29.4 (6.1)	29.8 (6.7)	0.07
**Smoking status**												
**Current**	834 (7.0%)	209 (7.6%)	0.02	91 (5.1%)	25 (6.5%)	0.06	367 (7.4%)	95 (7.3%)	0.00	285 (6.2%)	62 (6.3%)	0.00
**Former**	4,876 (40.9%)	1,047 (38.1%)	0.06	908 (50.6%)	164 (42.7%)	0.16	2,128 (43.0%)	517 (39.9%)	0.06	2,036 (44.4%)	388 (39.4%)	0.10
**Never**	6,209 (52.1%)	1,494 (54.3%)	0.04	795 (44.3%)	195 (50.8%)	0.13	2,453 (49.6%)	684 (52.8%)	0.06	2,267 (49.4%)	534 (54.3%)	0.10

© 2022 Parikh RB et al. *JAMA Network Open.*

	Hypertension			Congestive Heart Failure			Diabetes			Chronic Kidney Disease		
**Mean systolic blood pressure (SD)**	135.9 (13.8)	135.2 (13.5)	0.05	134.0 (14.4)	133.2 (14.4)	0.06	135.9 (13.8)	134.9 (13.8)	0.07	135.7 (13.9)	135.0 (14.1)	0.05
**Creatinine**	1.1 (0.4)	1.1 (0.3)	0.06	1.3 (0.5)	1.2 (0.5)	0.12	1.1 (0.4)	1.1 (0.4)	0.07	1.3 (0.5)	1.3 (0.5)	0.00
**ACE-inhibitors**	8,315 (69.2%)	1,976 (68.9%)	0.01	1,277 (70.7%)	286 (71.5%)	0.02	3,724 (74.9%)	955 (70.9%)	0.09	3,153 (68.4%)	684 (66.5%)	0.04
**Inpatient hospital admission**	1,770 (14.7%)	516 (18.0%)	0.09	673 (37.3%)	190 (47.5%)	0.21	801 (16.1%)	257 (19.1%)	0.08	1,066 (23.1%)	300 (29.2%)	0.14
**3 or more visits to emergency room**	934 (7.8%)	384 (13.4%)	0.18	403 (22.3%)	141 (35.3%)	0.29	468 (9.4%)	244 (18.1%)	0.26	572 (12.4%)	217 (21.1%)	0.23
**Number of PCP visits in past year**	4.3 (3.8)	5.2 (5.5)	0.21	5.6 (4.9)	7.9 (7.8)	0.41	4.8 (4.2)	6.3 (6.1)	0.32	4.8 (4.2)	6.2 (6.4)	0.30
**HbA1c**	6.7 (1.1)	6.9 (1.2)	0.13				7.0 (1.2)	7.0 (1.2)	0.05	6.9 (1.2)	7.0 (1.3)	0.13
**Charlson: Myocardial infarction**	1,055 (8.8%)	176 (6.1%)	0.10				531 (10.7%)	88 (6.5%)	0.15			
**Insulin**							1,031 (20.7%)	344 (25.5%)	0.11			
**Statins**							3,675 (73.9%)	959 (71.2%)	0.06			
**Charlson: Diabetes**				900 (49.9%)	202 (50.5%)	0.01				2,273 (49.3%)	555 (53.9%)	0.09
**Charlson: Chronic pulmonary disease**				540 (29.9%)	111 (27.8%)	0.05						
**Beta blockers**				1,412 (78.2%)	315 (78.8%)	0.01						
**Diuretics**				1,322 (73.2%)	329 (82.3%)	0.22						
**Ejection fraction**				50.9 (13.6)	49.0 (15.0)	0.14						

[a]MA = Medicare Advantage
[b]MSSP = Medicare Shared Savings Program
[c]SD = standard deviation
[d]IQR = interquartile range

© 2022 Parikh RB et al. *JAMA Network Open.*

eTable 5. Unadjusted and Adjusted Differences in Overall Spending Using HCCs Instead of Charlson Comorbidities

	Hypertension			Congestive Heart Failure			Diabetes			Chronic Kidney Disease		
	MA[a] (SD)	MSSP[b] (SD)[c]	Absolute Difference (95% CI)[d]	MA (SD)	MSSP (SD)	Absolute Difference (95% CI)	MA (SD)	MSSP (SD)	Absolute Difference (95% CI)	MA (SD)	MSSP (SD)	Absolute Difference (95% CI)
**Unadjusted**	$8,556 ($14,208)	$10,971 ($16,286)	$2,416 ($1,832, $3,032)	$15,832 ($19,923)	$19,437 ($20,479)	$3,605 ($1,426, $6,058)	$9,515 ($15,009)	$11,972 ($17,353)	$2,456 ($1,575, $3,408)	$11,125 ($16,885)	$13,727 ($17,737)	$2,603 ($1,539, $3,755)
**Adjusting for age, sex, Charlson**			$2,758 ($2,132, $3,420)			$5,924 ($3,265, $8,952)			$3,396 ($2,334, $4,554)			$3,119 ($1,957, $4,385)
**Adjusting for relevant claims-based characteristics**			$2,816 ($2,161, $3,512)			$6,441 ($3,633, $9,655)			$3,598 ($2,509, $4,786)			$3,213 ($2,004, $4,535)
**Adjusting for relevant claims and clinical characteristics**			$2,714 ($2,036, $3,436)			$5,846 ($2,959, $9,177)			$3,563 ($2,377, $4,867)			$3,237 ($1,958, $4,640)
**Adjusting for relevant claims, clinical characteristics, and SES**			$2,680 ($1,996, $3,409)			$5,959 ($3,059, $9,305)			$3,464 ($2,261, $4,790)			$3,321 ($2,025, $4,744)
**Propensity score adjusted**	$8,411 ($14,029)	$10,946 ($16,317)	$2,535 ($1,837, $3,280)	$15,158 ($19,721)	$19,518 ($20,776)	$4,360 ($1,850, $7,240)	$8,891 ($14,200)	$11,877 ($17,306)	$2,986 ($1,915, $4,163)	$10,770 ($16,601)	$13,755 ($17,967)	$2,985 ($1,658, $4,454)

[a]MA = Medicare Advantage
[b]MSSP = Medicare Shared Savings Program
[c]SD = standard deviation
[d]CI = confidence interval

© 2022 Parikh RB et al. *JAMA Network Open.*

eTable 6. Unadjusted and Adjusted Differences in Overall Spending (Charlson Comorbidities) Including MSSP Beneficiaries Without a Pharmacy Claim

	Hypertension			Congestive Heart Failure			Diabetes			Chronic Kidney Disease		
	MA[a] (SD)	MSSP[b] (SD)[c]	Absolute Difference (95% CI)[d]	MA (SD)	MSSP (SD)	Absolute Difference (95% CI)	MA (SD)	MSSP (SD)	Absolute Difference (95% CI)	MA (SD)	MSSP (SD)	Absolute Difference (95% CI)
**Unadjusted**	$8,566 ($14,264)	$10,593 ($16,218)	$2,027 ($1,533, $2,545)	$15,860 ($20,022)	$19,836 ($21,577)	$3,976 ($2,185, $5,945)	$9,527 ($15,071)	$11,638 ($17,531)	$2,110 ($1,405, $2,861)	$11,143 ($16,965)	$13,777 ($18,631)	$2,633 ($1,795, $3,526)
**Adjusting for age, sex, Charlson**			$1,843 ($1,345, $2,366)			$4,562 ($2,737, $6,566)			$2,333 ($1,586, $3,130)			$2,618 ($1,716, $3,583)
**Adjusting for relevant claims-based characteristics**			$1,911 ($1,395, $2,454)			$5,249 ($3,264, $7,441)			$2,531 ($1,739, $3,379)			$2,917 ($1,997, $3,902)
**Adjusting for relevant claims and clinical characteristics**			$2,304 ($1,661, $2,987)			$4,691 ($2,105, $7,649)			$3,173 ($2,054, $4,401)			$3,097 ($1,817, $4,504)
**Adjusting for relevant claims, clinical characteristics, and SES**			$2,284 ($1,631, $2,978)			$4,726 ($2,132, $7,694)			$3,051 ($1,924, $4,289)			$3,221 ($1,920, $4,652)
**Propensity score adjusted**	$8,645 ($14,286)	$10,641 ($16,301)	$1,996 ($1,457, $2,564)	$15,817 ($19,948)	$19,976 ($21,886)	$4,159 ($2,254, $6,265)	$9,389 ($14,831)	$11,438 ($17,334)	$2,049 ($1,220, $2,943)	$11,279 ($17,075)	$13,782 ($18,632)	$2,503 ($1,570, $3,503)
**Propensity score + coding adjusted**			$2,271 ($1,609, $2,976)			$5,302 ($2,685, $8,288)			$3,074 ($1,937, $4,324)			$3,074 ($1,768, $4,510)

[a]MA = Medicare Advantage
[b]MSSP = Medicare Shared Savings Program
[c]SD = standard deviation
[d]CI = confidence interval

© 2022 Parikh RB et al. *JAMA Network Open.*

eFigure 1. Practice Patterns among MSSP and MA Beneficiaries

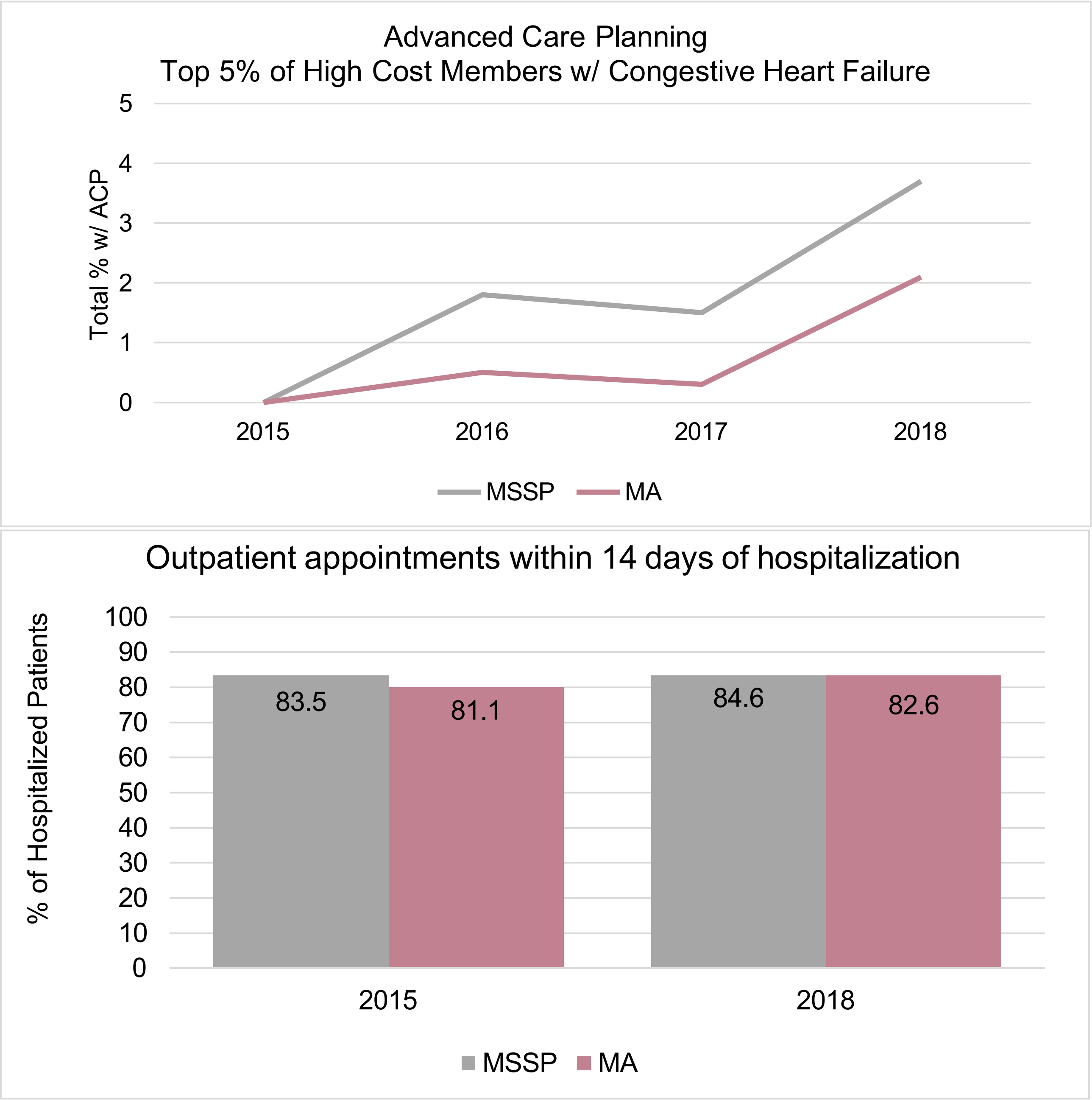

© 2022 Parikh RB et al. *JAMA Network Open.*

eFigure 2. Cohort Selection

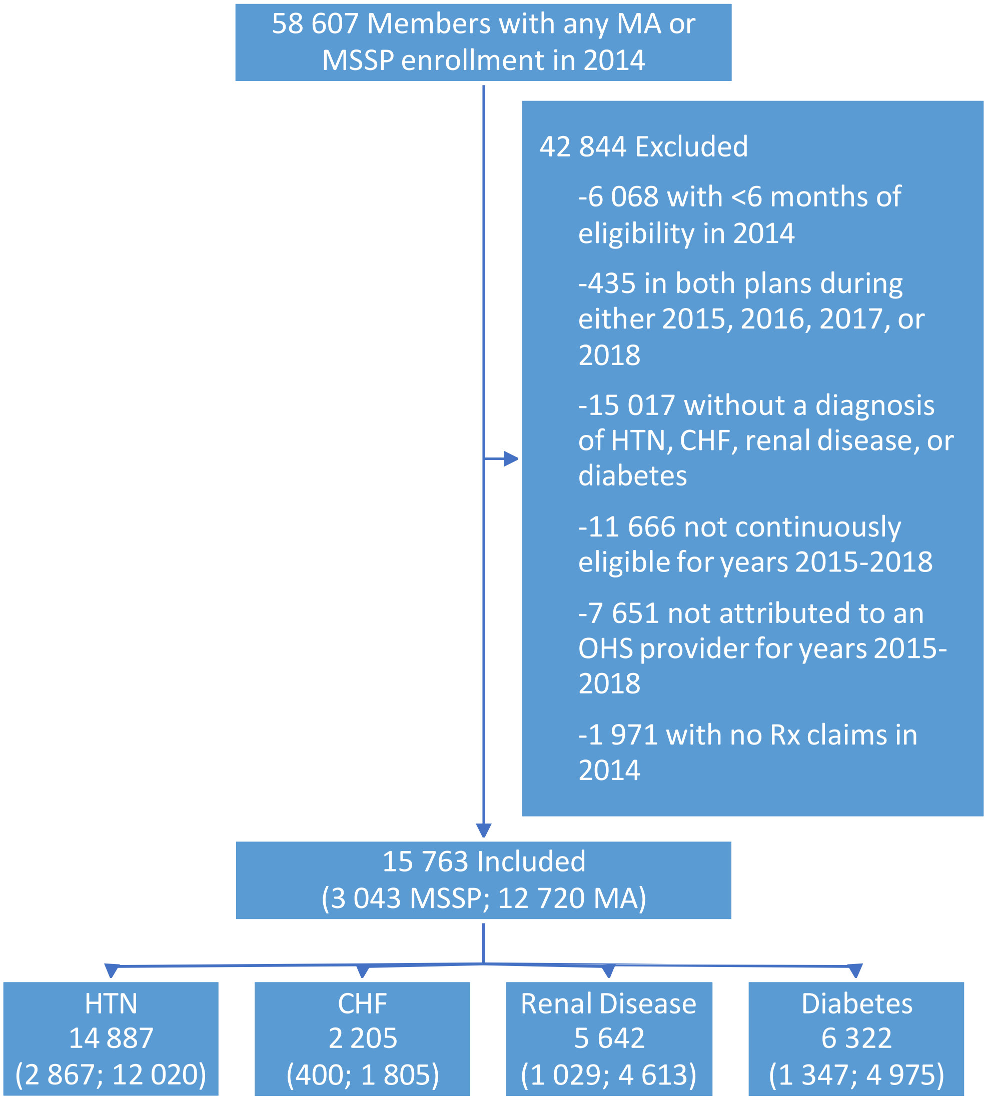

© 2022 Parikh RB et al. *JAMA Network Open.*

eFigure 3. Unadjusted and Adjusted Overall Spending in MSSP After Adjusting for Provider-Based Billing

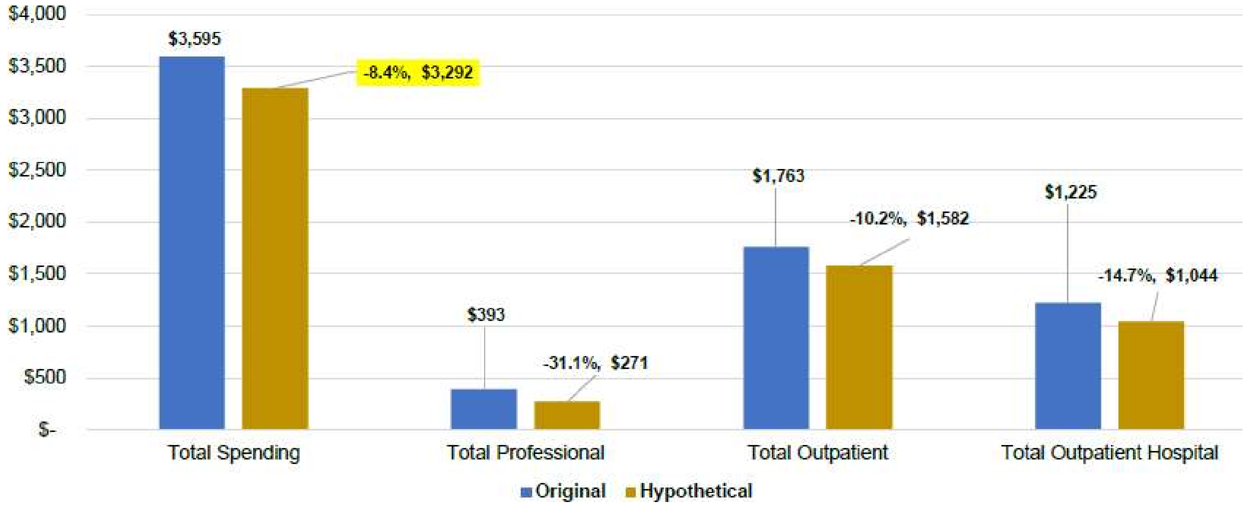

© 2022 Parikh RB et al. *JAMA Network Open.*

eFigure 4. Sensitivity Analysis of Changes in Differential Spending Over Time in MA and MSSP Cohorts, Excluding Decedents

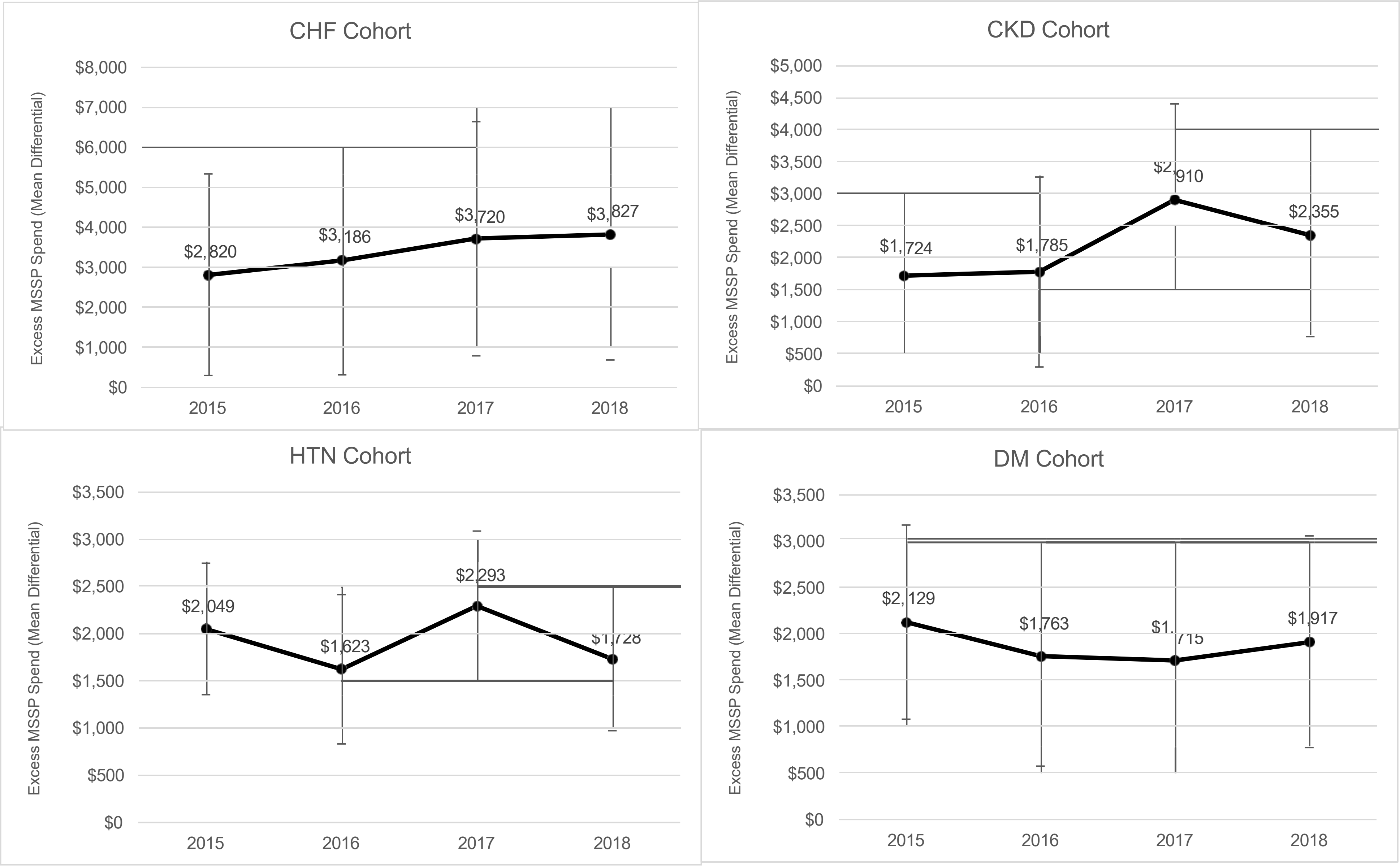

© 2022 Parikh RB et al. *JAMA Network Open.*

eFigure 5. Sensitivity Analysis of Changes in Differential Spending Over Time in MA and MSSP Cohorts, Excluding Dual-Eligible Beneficiaries

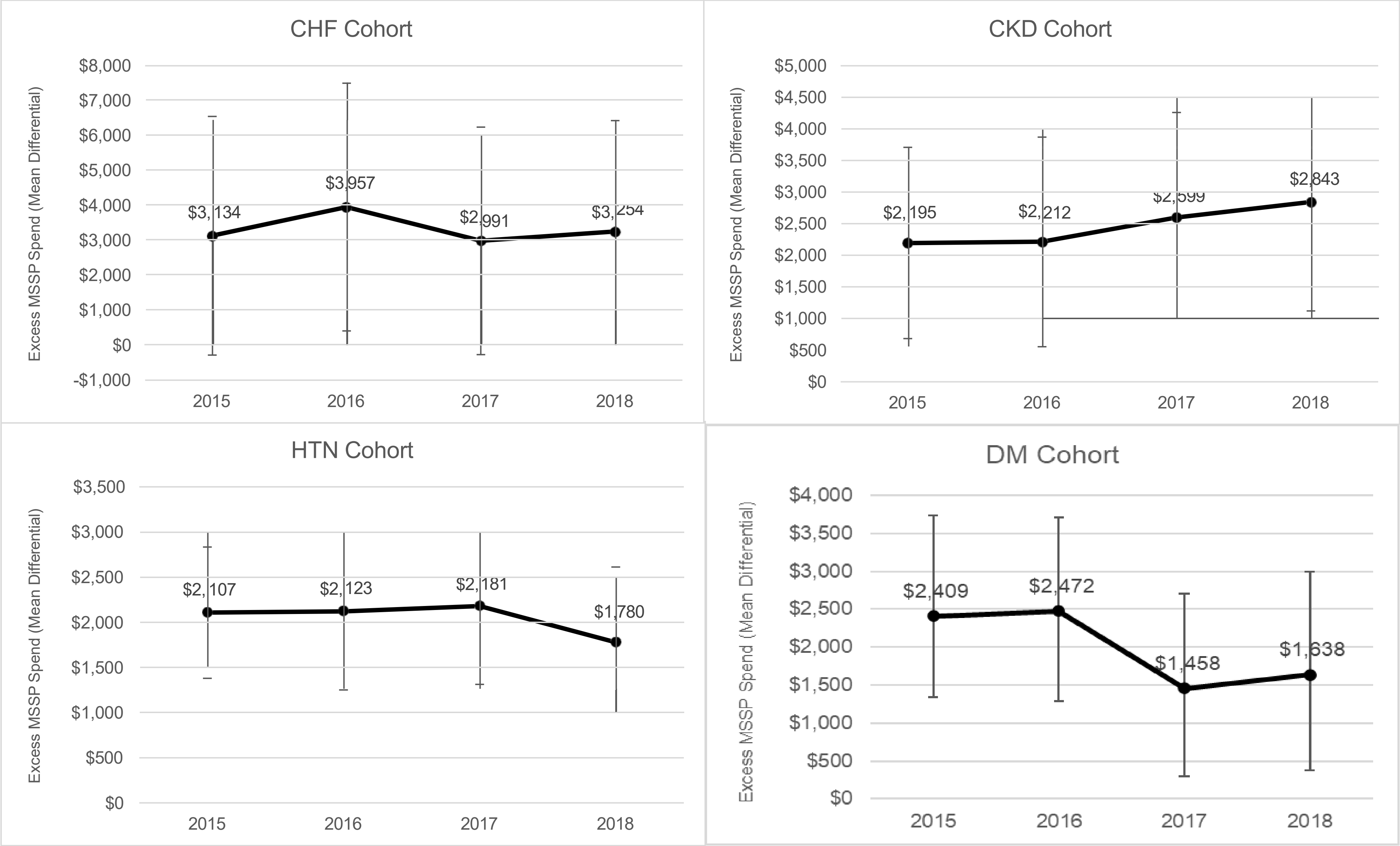

© 2022 Parikh RB et al. *JAMA Network Open.*

eFigure 6. Sensitivity Analysis of Changes in Differential Spending Over Time in MA and MSSP Cohorts, Using Intent-to-Treat Approach

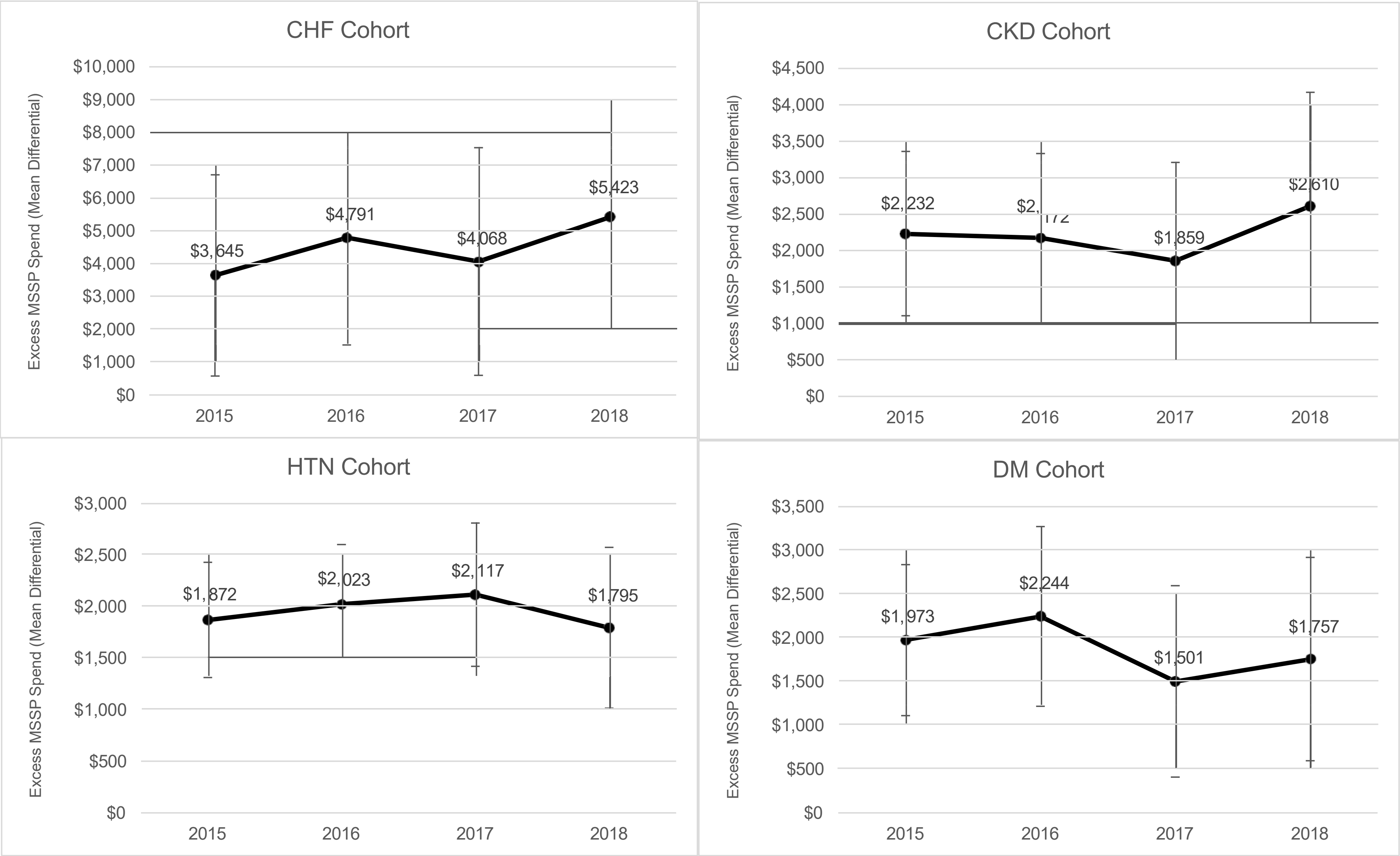

© 2022 Parikh RB et al. *JAMA Network Open.*
